# Average surface flows before the formation of solar active regions and their relationship to the supergranulation pattern

**DOI:** 10.1051/0004-6361/201935591

**Published:** 2019-08-02

**Authors:** A. C. Birch, H. Schunker, D. C. Braun, L. Gizon

**Affiliations:** 1 Max-Planck-Institut für Sonnensystemforschung, Justus-von-Liebig-Weg 3, 37077 Göttingen, Germany; 2 NorthWest Research Associates, 3380 Mitchell Lane, Boulder, CO 80301, USA; 3 Institut für Astrophysik, Georg-August-Universität Göttingen, Friedrich-Hund-Platz 1, 37077 Göttingen, Germany

**Keywords:** Sun: activity, Sun: helioseismology, Sun: magnetic fields, sunspots

## Abstract

**Context.:**

The emergence of solar active regions is an important but poorly understood aspect of the solar dynamo.

**Aims.:**

Knowledge of the flows associated with the rise of active-region-forming magnetic concentrations through the near-surface layers will help determine the mechanisms of active region formation.

**Methods.:**

We used helioseismic holography and granulation tracking to measure the horizontal flows at the surface that precede the emergence of active regions. We then averaged these flows over about sixty emerging active regions to reduce the noise, selecting active regions that emerge into relatively quiet Sun. To help interpret the results, we constructed a simple model flow field by generating synthetic “emergence locations” that are probabilistically related to the locations of supergranulation-scale convergence regions in the quiet Sun.

**Results.:**

The flow maps obtained from helioseismology and granulation tracking are very similar (correlation coefficients for single maps around 0.96). We find that active region emergence is, on average, preceded by converging horizontal flows of amplitude about 40 ms^−1^. The convergence region extends over about 40 Mm in the east-west direction and about 20 Mm in the north-south direction and is centered in the retrograde direction relative to the emergence location. This flow pattern is largely reproduced by a model in which active region emergence occurs preferentially in the prograde direction relative to supergranulation inflows.

**Conclusions.:**

Averaging over many active regions reveals a statistically significant pattern of near-surface flows prior to emergence. The qualitative success of our simple model suggests that rising flux concentrations and supergranule-scale flows interact during the emergence process.

## 1. Introduction

Solar active regions are thought to be the result of magnetic flux concentrations rising from the base of the convection zone (Spiegel & Weiss 1980). Alternative scenarios are that these magnetic flux concentrations are formed throughout the convection zone (Nelson et al. 2013) or in the near-surface layers (Brandenburg 2005). Determining the origin of solar active regions would place an important constraint on models of the solar dynamo (see Charbonneau 2014 for a recent review).

Local helioseismology, which is the use of solar oscillations to study the solar interior in three dimensions (Gizon & Birch 2005; Gizon et al. 2010), has been applied extensively to search for subsurface signatures of magnetic flux concentrations rising through the solar interior prior to the formation of active regions. Numerous case studies of the emergence of individual active regions have been carried out (see the introduction of Birch et al. 2013 for references). As discussed by Birch et al. (2013), case studies have not provided a consensus helioseismic picture of the subsurface changes associated with the preemergence stage of active region formation. The situation has not changed since then (see also Komm et al. 2015 for a more recent overview).

Statistical analysis is a necessary next step in addition to case studies. Statistical approaches are valuable as they can potentially uncover helioseismic signals that are too weak to be found for single active regions. Based on the models for rising flux tubes from Fan (2008), Birch et al. (2010) showed that the dominant helioseismic signal is expected to be caused by the ≈100 ms^−1^ horizontal flows associated with a rising flux tube. The helioseismic signals expected to result from these flows are, however, too weak to be seen for a single active region. Birch et al. (2010) suggested that a study of about one hundred active regions would be necessary for a statistically significant measurement of the subsurface flows associated with a rising flux concentration.

Statistical observational studies have indeed shown that the helioseismic signatures associated with the formation of active regions are weak. Birch et al. (2013) applied helioseismic holography to observations obtained by the Global Oscillation Network Group (GONG; Harvey et al. 1998) to search for flows preceding the formation of the one hundred active regions selected for helioseismic study by Leka et al. (2013). They found statistically significant near-surface flows of about 15 ms^−1^ within about 30 Mm of the emergence location during the day preceding the formation of active regions. The geometry of these flows suggested a converging flow toward the emergence location. Using the same active regions and the same helioseismic measurements, Barnes et al. (2014) showed that the single measurement that best distinguishes emerging active regions from quiet-Sun control regions, even one day before emergence, is the surface magnetic field. In addition, Barnes et al. (2014) found small differences in the probability distributions of both north-south flows and radial vorticity for preemergence regions and quiet-Sun regions that later did not show flux emergence.

The helioseismic signatures during the growth phase of active regions are also weak. Komm et al. (2011) used ring-diagram analysis (Hill 1988) with a horizontal spatial resolution of 15° (≈180 Mm) to measure the subsurface flows associated with more than 800 active regions. From this sample, they selected the most rapidly growing 20% of the regions and determined the average horizontal and vertical flows associated with these regions. They found ≈5 ms^−1^ prograde flows and ≈0.4 ms^−1^ vertical flows associated with the growth of the active regions.

In an extension of the approach of Leka et al. (2013), Schunker et al. (2016) identified a sample of about one hundred emerging active regions observed by the Helioseismic and Magnetic Imager on board the Solar Dynamics Observatory (SDO/HMI). Each emerging active region was assigned a quiet-Sun control region that had the same disk position, but was observed at a different time (in almost all cases within ten days). The SDO/HMI observations are an improvement over the GONG and Michelson Doppler Imager (MDI) observations used by Birch et al. (2013) as they have higher spatial resolution and thus allow helioseismology closer to the limb, which in turn allows measuring flows further back in time before the emergence time. Another advantage is that HMI magnetograms are available at 45 s cadence, unlike the (typically available) 96 min magnetograms from MDI; this enables the determination of more precise emergence times.

Birch et al. (2016) used helioseismic holography and local correlation tracking to measure the surface flows associated with the emergence of the active regions identified by Schunker et al. (2016). Birch et al. (2016) then compared the surface flows with the surface flows in various simulations of rising flux tubes (following the setup of Cheung et al. 2010; Rempel & Cheung 2014). This comparison showed that the simulations where the rise speed of the flux tube at a depth of 20 Mm is more than about 150 ms^−1^ produce preemergence diverging flows that are not consistent with the observations. Norton et al. (2017) found from HMI vector magnetograms that the surface magnetic flux increases during the emergence process more slowly than predicted by simulations of flux emergence with rise speeds of 500 ms^−1^ at 20 Mm. Norton et al. (2017) suggested that these observations imply that the rise speed is too high in the simulations. There are, however, case studies that suggest very high rise speeds for some individual active regions. As one example, Kosovichev et al. (2018) measured an upward pattern motion of the horizontal divergence (inferred from time-distance helioseismology) of about 1.3 km s^−1^ at 20 Mm for AR11726, which emerged in April 2013. This particular active region was not in the sample used by Schunker et al. (2016), which covered the time period May 2010 to November 2012.

Here we extend the work of Birch et al. (2016) and measure the spatial variations and temporal evolution of near-surface flows before and during the emergence of the active regions described by Schunker et al. (2016). Our goal is to determine whether statistically significant flow patterns are associated with the emergence of active regions. We expect that these flows will be useful for constraining models for the origin and formation of active regions.

## 2. Data reduction

The catalog of Schunker et al. (2016) describes 105 active regions that emerge on the visible disk. It also contains an associated set of quiet-Sun control regions that have the same latitude and distance from the central meridian. Schunker et al. (2016) assigned each active region a *P*-factor that describes the amount of preexisting flux near the emergence location. The *P*-factors are determined by examining line-of-sight magnetograms by eye. A *P*-factor larger than two indicates emergence into an area with preexisting magnetic field. Here we only consider emerging active regions with a *P*-factor of two or lower. These emerging active regions and their partner quiet-Sun control regions form the basis for the data analysis carried our here. We refer to the emerging active regions as “AR” and to the quiet-Sun control regions as “QS” regions.

For each AR and associated QS region there is a set of SDO/HMI Dopplergrams, continuum intensity images, and line-of-sight magnetic field observations as described by Schunker et al. (2016). The data cubes of Dopplergrams were used as input for the helioseismic holography (Sect. 2.1). The data cubes of continuum intensity images were used as input to a local correlation tracking algorithm (Appendix B). The magnetograms were used to determine the emergence location and emergence time, and to classify the complexity of the emergence (as described in Schunker et al. 2016).

### 2.1. Helioseismic holography

We used surface-focusing holography as described in Birch et al. (2016). The input data were Doppler data cubes of length 6.825 h (547 images). Each Dopplergram was remapped using a Postel projection, with the center of the projection given in Table A.1 of Schunker et al. (2016). Each resulting map had a grid spacing of 1.39 Mm and contained 512 × 512 grid points. The coordinates in the remapped images are *x* and *y*, with *x* increasing westward (prograde direction) and *y* increasing northward. The data cubes were then filtered using the phase-speed filter 3 from Couvidat et al. (2005). This phase-speed filter selects waves with a lower turning-point depth of about 3 Mm. Surface-focusing holography with a pupil size matched to the target phase speed (see table in Couvidat et al. 2005) was then applied to each data cube to measure east minus west, north minus south, and in minus out travel-time differences. After computing travel-time maps, we applied a filter to reduce the contribution of realization noise to the travel-time maps. The filter had a value of one for angular degree *kR*_⊙_ < 140, was zero for *kR*_⊙_ > 220, and had a raised cosine taper in between. After the filtering, the east-west and north-south travel-time maps were calibrated to units of ms^−1^ using the method of Birch et al. (2016). We refer to these calibrated travel-time maps as *v*_*x*_ (westward flow, positive for prograde flows) and *v*_*y*_ (northward flow, positive for flows to the north). The horizontal vector velocity *v*_h_ is given by (*v*_*x*_, *v*_*y*_). We removed large-scale field effects by subtracting the best-fit second-order polynomial in the *x* and *y* pixel coordinates from each map. This process also removes true large-scale flows (differential rotation, meridional flow); the focus in the current work is the local flows associated with the emergence process.

### 2.2. Data selection

The noise level in helioseismic holography depends mainly on the duty cycle and the distance from the central meridian. Here we chose to only use the time intervals when the duty cycle for the Dopplergrams and intensity images (used for the local correlation tracking, Appendix B) were both above 90% and the central meridian distance was smaller than 50°. Appendix C provides the motivation for these thresholds. The sample of Schunker et al. (2016) contains 65 regions with a *P*-factor of two or lower that at three hours before emergence satisfy these requirements. This number decreases as the time before or after emergence increases (mostly because of the restriction on distance from the central meridian).

## 3. Ensemble average flow maps

**Fig. 1.**
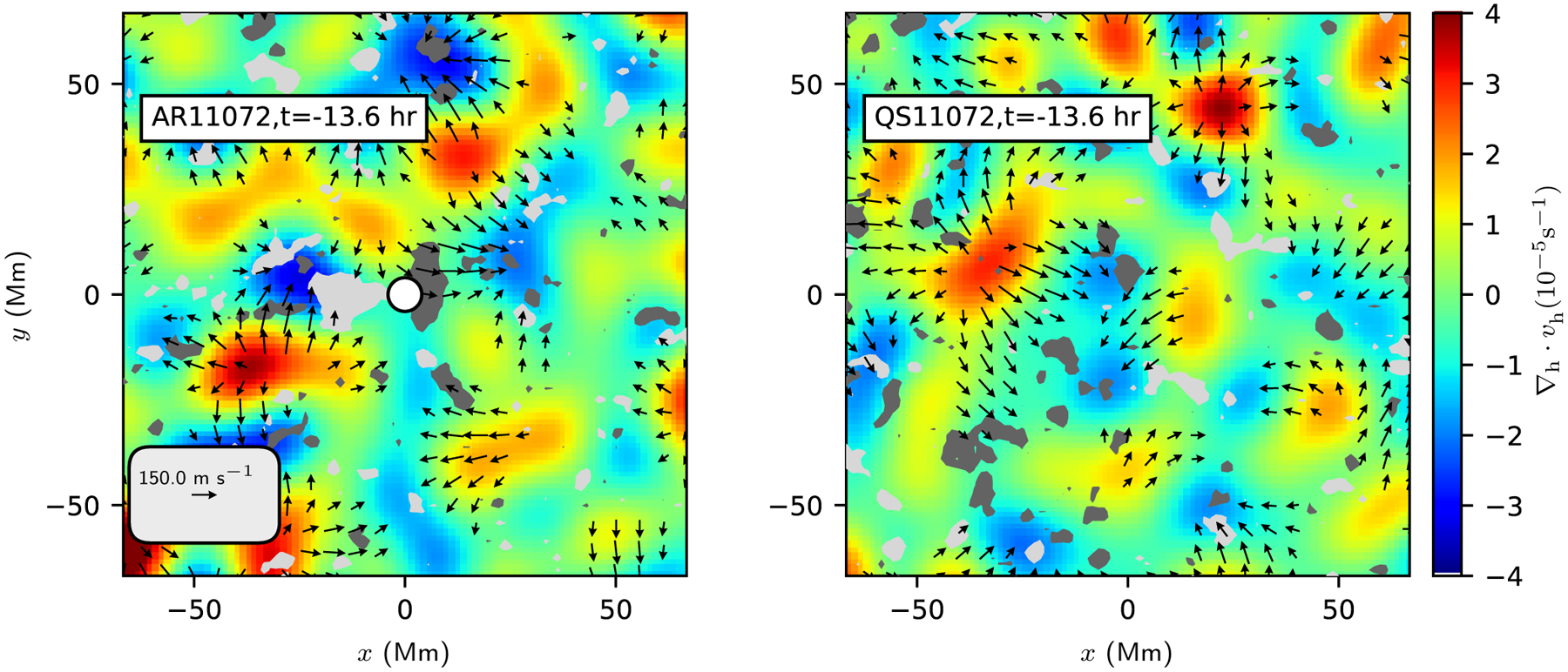
Example helioseismology maps of the surface horizontal flow and line-of-sight magnetograms for a single emerging active region (AR11072; *left*) at 13.6 h before emergence and the corresponding quiet-Sun region (*right*). The emergence location is shown by the white circle at the origin in the *left panel*. The maps show the horizontal divergence (colors; blue for converging flows and red for diverging flows), the horizontal flows estimated from the calibrated travel times (black arrows), and the line-of-sight component of the magnetic field (light and dark gray for the two polarities; only line-of-sight field stronger than 10 G is shown). The *x* and *y* components of the flows each have an rms of about 60 ms^−1^. The dominant features in both maps are the diverging horizontal flows associated with supergranules. This example shows no clear precursor flows before the emergence time. The scale arrow in the lower left corner of the first panel shows a flow of 150 ms^−1^. Flows weaker than 75 ms^−1^ are not shown in these maps. The *x* -coordinate increases westward (prograde direction) and *y* increases northward.

Figure 1 shows the horizontal near-surface flows measured from helioseismology for an example emerging active region and an example quiet-Sun control region. In both cases, the most apparent flows are the diverging flows that are associated with the supergranulation pattern. The largest flows have amplitudes of about 250 ms^−1^ and the *v*_*x*_ and *v*_*y*_ components of the flows each have an rms of about 60 ms^−1^. In this example, no flow pattern can be unambiguously identified as associated with the emerging active region; this is consistent with Birch et al. (2013).

The absence of clear preemergence flow patterns in individual active regions motivated us to consider the flows associated with emerging active regions from a statistical point for view. Here we average the individual flow maps over all of our emerging active regions (Sect. 2). As a control, we carry out the same averaging procedure for the quiet-Sun sample as well.

The averaging process is as follows. The first step is to determine the emergence location. We used the method of Birch et al. (2016) and defined the emergence location for each active region as the centroid of the pixels where the change in the line-of-sight magnetic field from 24 h before emergence to 8 h after emergence was more than 30% of the maximum change. For the special case of AR11456, a small emergence near a preexisting field, we used the magnetic field at 24 h (rather than 8 h) after emergence in the calculation because this produced a better (by eye) emergence location. After computing the emergence location, we shifted each map so that the emergence location was at position (*x*, *y*) = (0, 0). We then flipped regions in the southern hemisphere in the north-south direction and flipped the sign of both the line-of-sight magnetic field and the *y*-component of the velocity. This processes accounts for Joy’s law and allows averaging over regions in both hemispheres. The coordinate system in these aligned (and sometimes flipped) maps is such that *x* increases in the direction of rotation and *y* increases away from the equator. Flows with positive *v*_*y*_ are directed poleward. We followed Schunker et al. (2016) and defined the emergence time as the time when the total unsigned flux reaches 10% of the maximum unsigned flux seen within 36 h of the time when the region was assigned a NOAA active region number (see also Leka et al. 2013, for a discussion of this definition). After determining an emergence time for each active region, we averaged all of the maps at a fixed temporal o set from the emergence time.

**Fig. 2.**
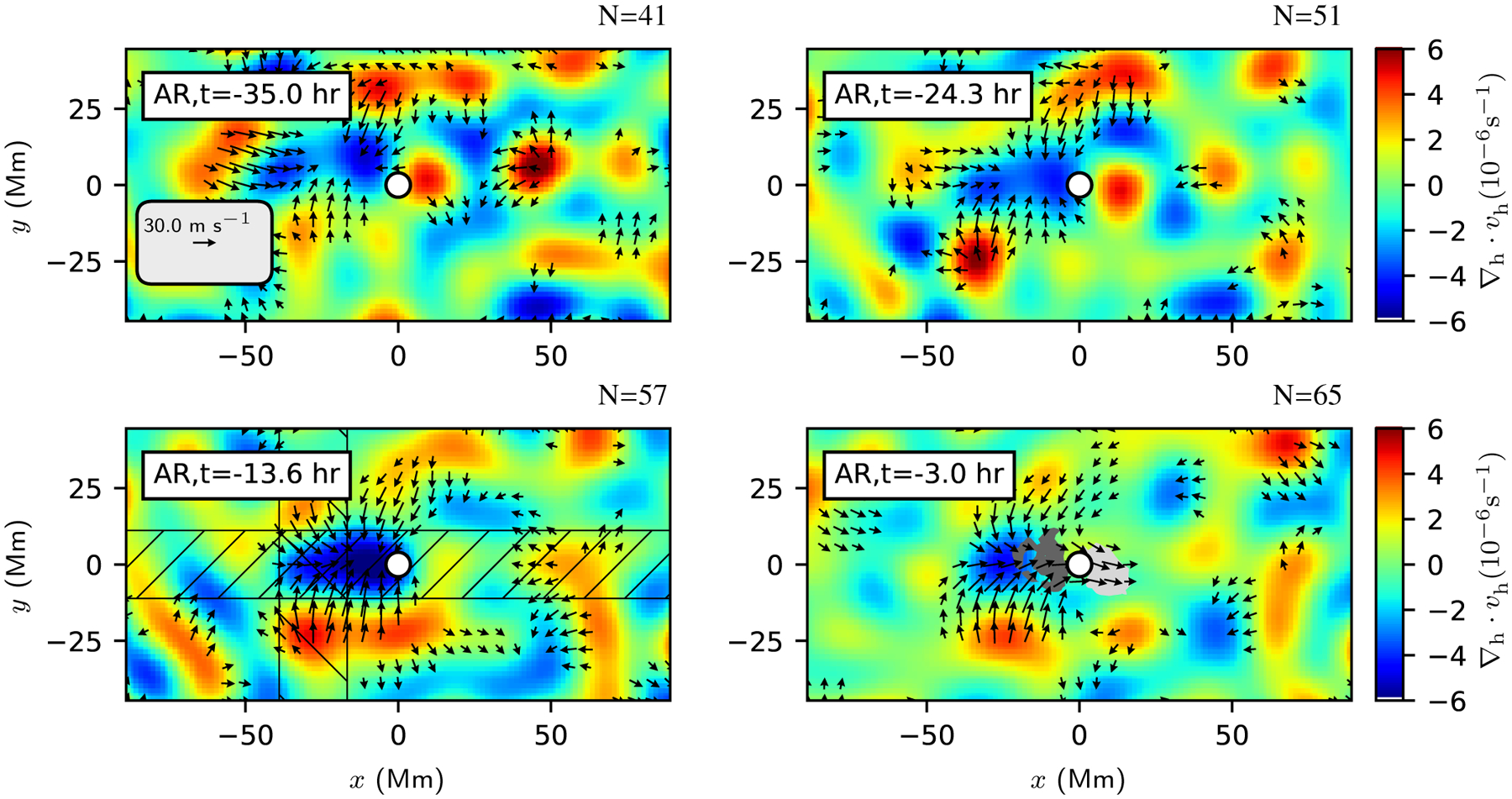
Helioseismology flow maps and magnetograms after averaging over all emerging active regions from Schunker et al. (2016) with a *P*-factor of two or lower. The small white circle at (*x*, *y*) = (0, 0) shows the emergence location. Time increases from 35 h before emergence (*top left*) to 3 h before emergence (*bottom right*). The number *N* of regions contributing to each average map is shown at the *top right* of each panel. As in Fig. 1, the black arrows show horizontal flows measured from helioseismic holography and the colors show the horizontal divergence (red for diverging flows and blue for converging flows). The gray shaded regions show where the average line-of-sight magnetic field exceeds 30 G (light and dark gray show the two polarities). The scale arrow in the *top left panel* shows a prograde flow of 30 ms^−1^; flows weaker than 15 ms^−1^ are not shown. By 24 h before emergence, an east-west aligned converging flow of about 20 ms^−1^ is located to the east (retrograde direction) of the emergence location. The hatched regions in the panel for *t* = −13.6 h show the averaging regions for the cuts shown in Figs. 3 and 4. The noise level varies from about 10 to about 8 ms^−1^ in the *x* and *y* components of the flow as *N* increases.

**Fig. A.1.**
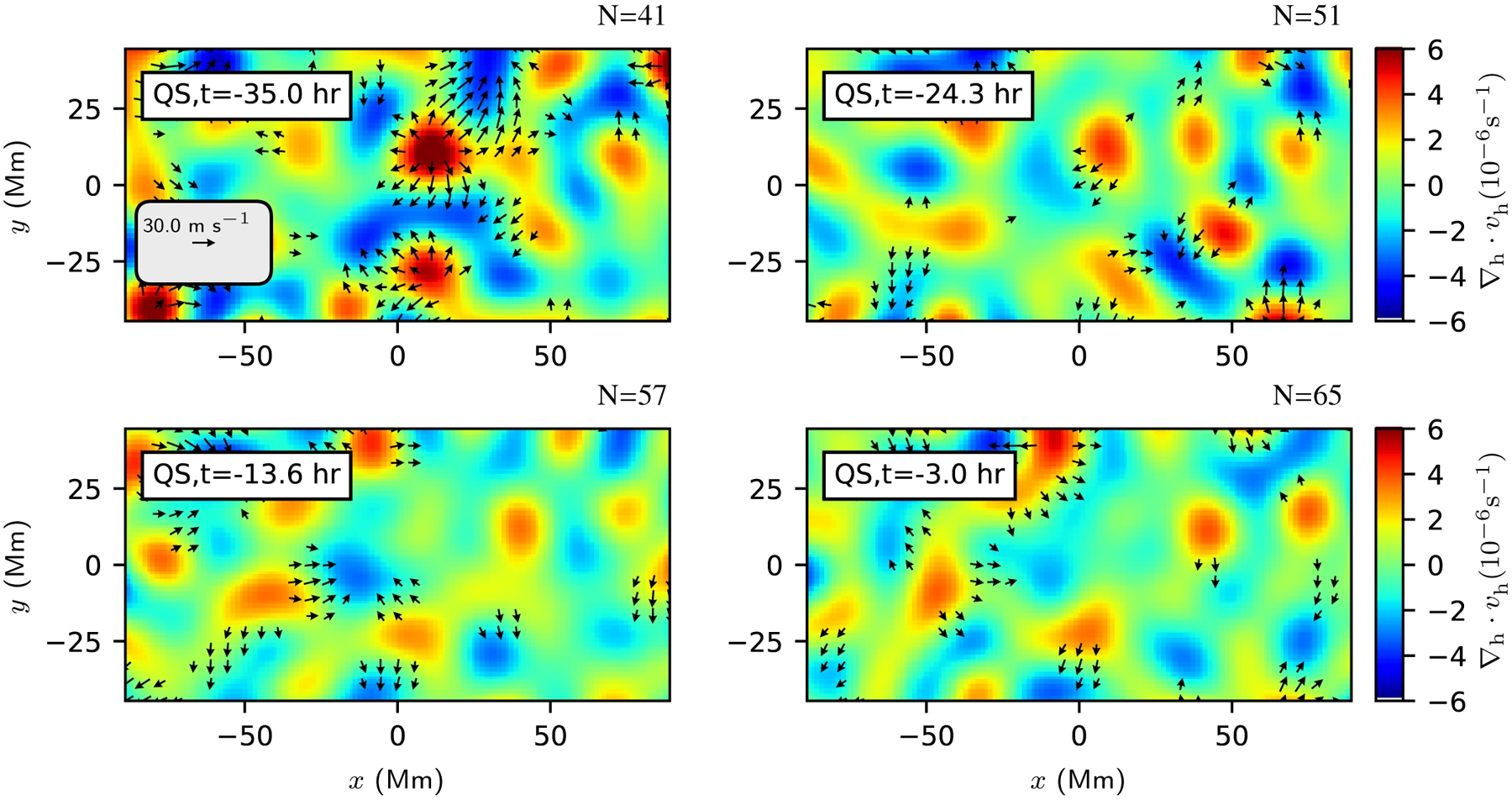
Quiet-Sun averages shown in the same format as Fig. 2. There is no apparent pattern in these flow maps.

**Fig. C.2.**
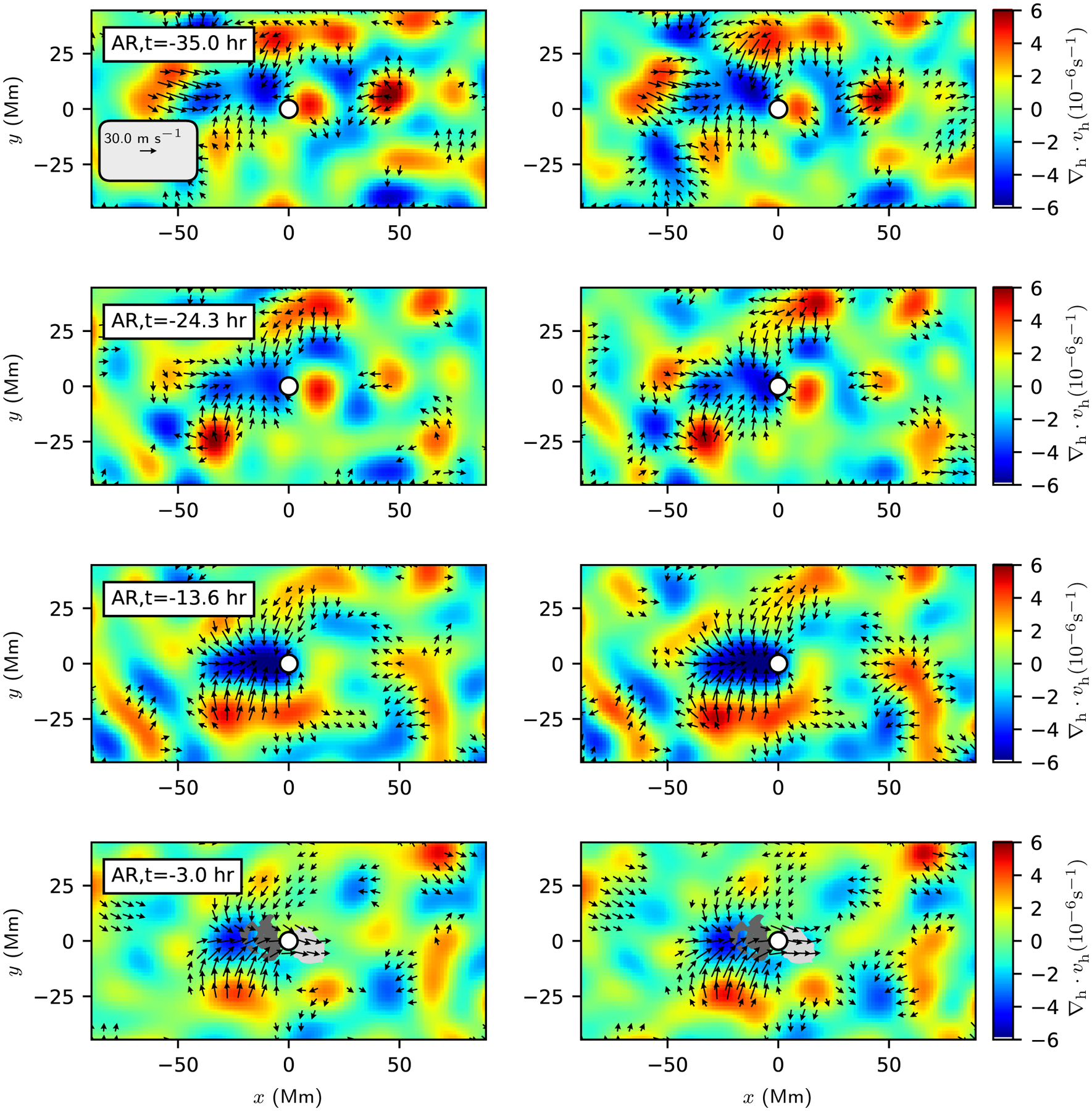
Flow maps from helioseismology (*left*) and LCT (*right*) after averaging over all emerging active regions. The format is the same as in Fig. 2.

Figure 2 shows the resulting average helioseismic flow maps for the emerging active regions. The number of regions included in the average varies from *N* = 41 at 35 h before emergence to *N* = 65 at 3 h before emergence. As discussed in Sect. 2.2, the main effect is the limit on distance from the central meridian (for any particular AR, further before emergence means farther east). If we instead use the common set of regions that is available from 35 h to 3 h before emergence, we obtain similar flow patterns, but they have a higher noise level. At 35 h before emergence, the active region averages do not show any obvious structure. The rms amplitude of the flows is about 10 ms^−1^ for both the *x* and *y* component of the flows (roughly consistent with the 60 ms^−1^ rms in the individual maps and an average over about 40 maps). At 24 h before emergence, a weak converging flow (about 20 ms^−1^) is located east (retrograde direction) of the emergence location. This flow is also seen in the average flow maps from the local correlation tracking (Fig. C.2). The presence of a converging flow is qualitatively consistent with the results of Birch et al. (2013). The physical origin of this converging flow is not known; we explore a simple model in Sect. 4. In the corresponding quiet-Sun flow maps (Fig. A.1) we do not see a corresponding feature; these maps are consistent with “noise” due to supergranulation flows (the rms for each component of the flows is about 8 ms^−1^, which is roughly $60{\text{ms}}^{-1}/\sqrt{N}$, where *N* = 57 is the number of maps used in the average).

At *t* = −13.6 h, the converging flow in the AR average has increased in strength to roughly 40 ms^−1^. The flow is extended in the east-west direction and centered to the east (retrograde direction) of the emergence location. At this time there is also a prograde flow (about 20 ms^−1^) to the east of the emergence location. This prograde flow is qualitatively similar to what was seen by Birch et al. (2013).

At three hours before the emergence time, a bipole feature is seen in the average line-of-sight magnetic field. The definition of emergence time that we have applied here (from Schunker et al. 2016) allows a magnetic field before the emergence time (see Schunker et al. 2016 for more discussion of this point). There is still a converging flow and a prograde flow to the east of the emergence location. The prograde flow now extends into the leading polarity. The average flow map at three hours before emergence is qualitatively consistent with the flow pattern shown in Fig. 5 of Birch et al. (2013), although the flows here are somewhat stronger (40 ms^−1^ here compared to roughly 10 ms^−1^ from Birch et al. 2013). This is presumably due to the improved spatial resolution of the measurements shown here.

**Fig. 3.**
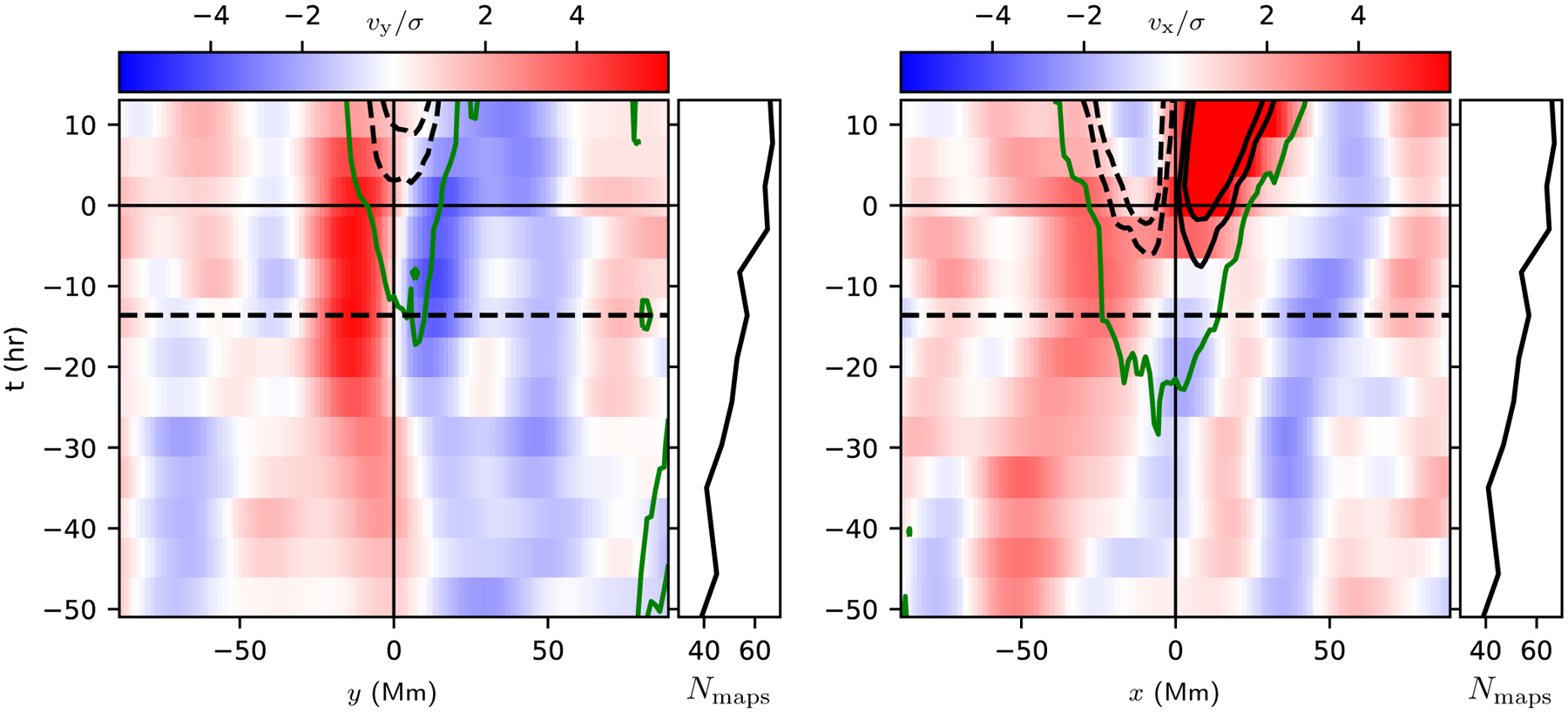
Time evolution of horizontal surface flows after averaging over the AR. *Left panel*: *v*_*y*_ as a function of *y* after averaging over a strip of half-width 11.1 Mm centered on *x* = −28 Mm. *Right panel*: *v*_*x*_ as a function of *x* after averaging over a strip of half-width 11.1 Mm centered on *y* = 0. The averaging regions are shown as hatched regions in the *bottom left panel* of Fig. 2. In both cases the velocities are scaled by the error estimated from the scatter observed in the quiet-Sun control regions. The black lines show contours of the average line-of-sight magnetic field, and the dashed line shows negative contours. The spacing between contours is 10 G, and the first contours are at ±20 G. The green lines show 10 G contours of the average unsigned line-of-sight magnetic field. The black dashed lines at *t* = −13.6 h show the time corresponding to the map shown in the *lower left panel* of Fig. 2 and also to the slices shown in Fig. 4. The numbers of maps that contribute to the average at each time are shown by the black curves to the right of each panel.

Figure 3 shows the time evolution of slices through *v*_*y*_ and *v*_*x*_ after averaging over ±11.1 Mm (±8 pixels) in the transverse direction (see the lower left panel of Fig. 2 for the averaging regions; these regions are selected to capture the converging flow in the east-west cut and the converging flows around *y* ≈ ±25 Mm in the north-south cut). The north-south converging flow can be see from about 24 h before emergence and lasts into the emergence phase. There is a suggestion of a prograde flow located to the east (retrograde direction) of the emergence location. After the emergence time, there is prograde velocity in the leading polarity and retrograde velocity in the trailing polarity (see Schunker et al. 2019, for a detailed study of the post-emergence stage).

**Fig. 4.**
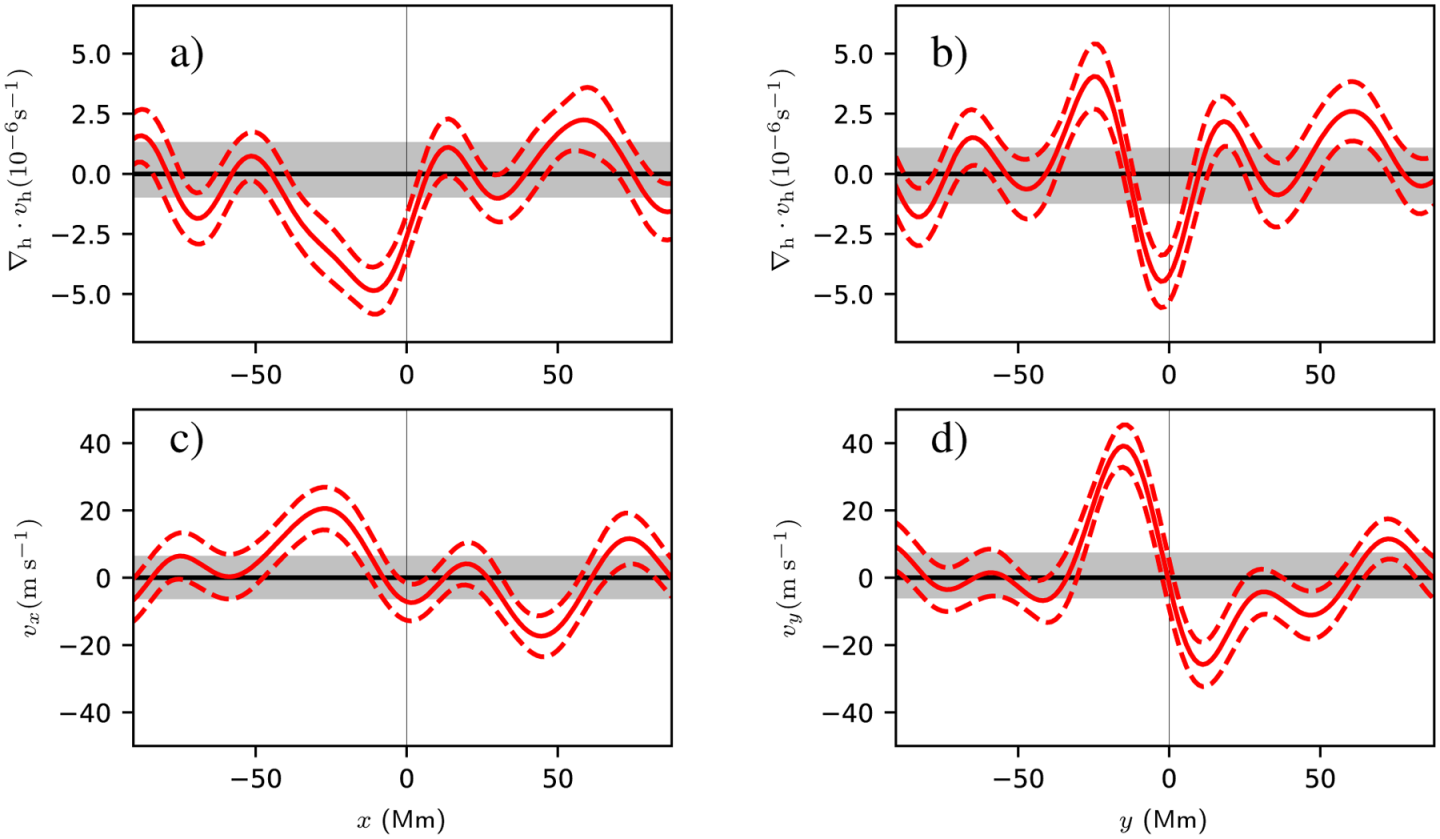
Slices through the horizontal divergence (*top row*), *v*_*x*_ (*bottom left*), and v_*y*_ (*bottom right*) at *t* = −13.6 h for the AR. The red solid lines show the average values for the AR, and the red dashed lines show the associated 1*σ* error in the mean. The gray shaded regions show the 1*σ* error estimates from the quiet-Sun control regions. *Left column*: slices are taken at *y* = 0. *Right column*: slices are taken at *x* = −28 Mm. In all cases the divergence or flow component has been averaged over ±11.1 Mm (8 grid points) in the transverse direction. The averaging regions for the cuts are shown in the *bottom left panel* of Fig. 2.

Figure 4 shows slices through the flow field at *t* = −13.6 h. The preemergence north-south converging flow at this time has an amplitude of about 40 ms^−1^ and stands out clearly above the noise level of about 8 ms^−1^. The *v*_*x*_ flow has a peak amplitude of about 20 ms^−1^ and is barely above the noise level. The slices through the horizontal divergence show the converging flow to the east (retrograde direction) of the emergence location.

## 4. Flows implied by a simple model for emergence locations at *t* = −13.6 h

The dominant features seen in the flows maps for individual emerging active regions are supergranules. It has been suggested that the locations where active regions emerge are correlated with the supergranulation pattern (e.g., Bumba & Howard 1965; Howard et al. 1979), although there is not agreement on this point (e.g., Zirin 1974). If this were the case, the average flow maps (Fig. 2) would show features due to the (perhaps only partially) coherent averaging over the flows that are associated with the supergranulation. In this section, we explore the idea that the flow pattern associated with emergence at *t* = −13.6 h (Sect. 3) can be understood as a consequence of a correlation between the locations of flux emergence and the supergranulation pattern. We chose to focus on this time period because the observed flow pattern is clear but the surface magnetic field is still weak.

Here we take the approach of using the quiet-Sun control regions together with a Monte Carlo generation of synthetic “emergence locations” to predict what we would expect to see if emergence locations had a particular relationship to the supergranulation pattern. The average flow maps at *t* = −13.6 h before emergence (Fig. 2) suggest that the emergence locations are on average to the west of a (supergranulation-scale) converging flow.

**Fig. 5.**
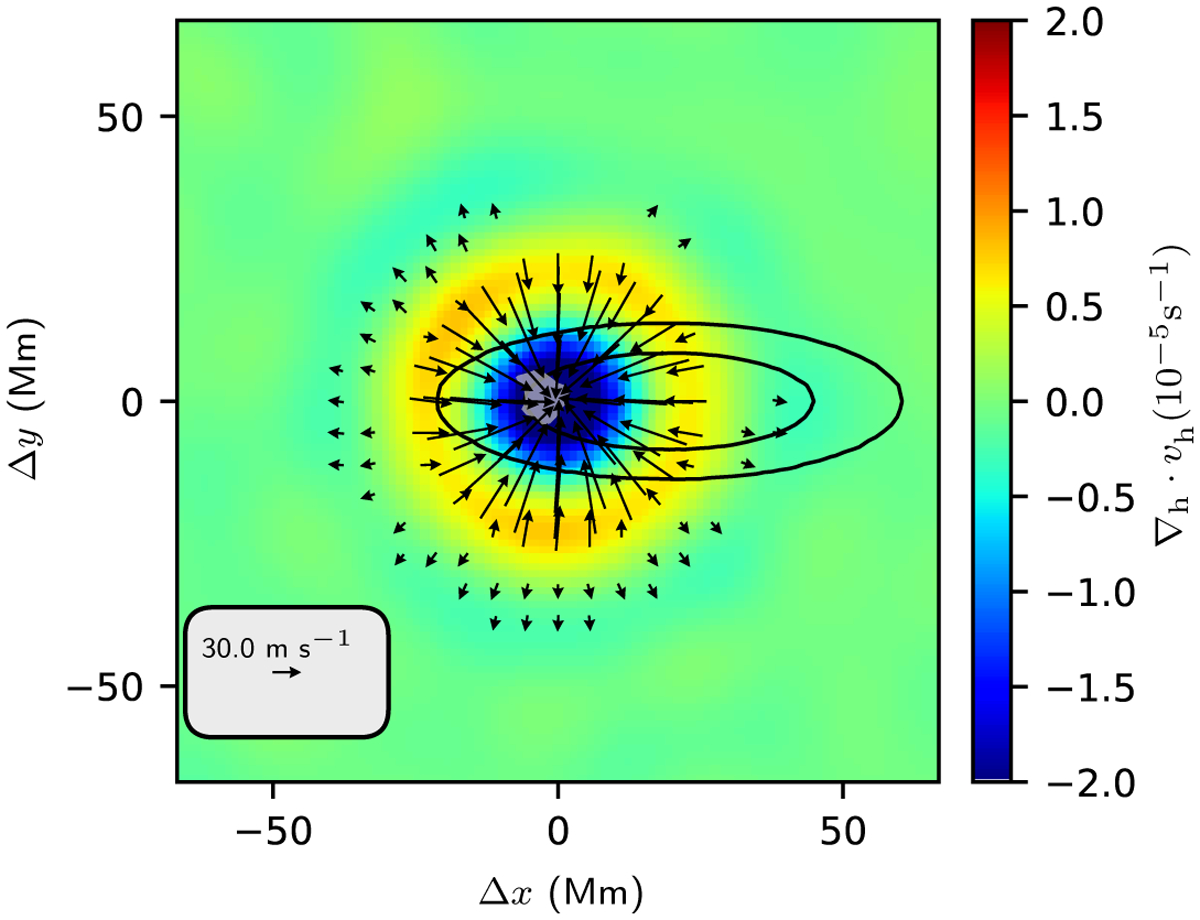
Average over 1129 supergranulation-scale convergence regions and the probability distribution function used to generate synthetic “emergence locations”. These synthetic emergence locations are used in a simple model for the flows at *t* = −13.6 h before emergence (Fig. 6). The colors in the background show the horizontal divergence (blue for converging flows and red for diverging flows), and the black arrows show the horizontal flow. Flows weaker than 15 ms^−1^ are not shown. The gray shaded region shows where the average unsigned magnetic field exceeds 10 G. The black curves are the contours of the probability distribution of synthetic emergence locations that enclose 68% and 95% of the probability.

As a first step, we identified the supergranulation-scale convergence features in the QS regions at *t* = −13.6 h. The algorithm is described in Appendix D. Figure 5 shows the average of the 1129 features that were identified by this algorithm. The flow pattern consists of a core with horizontally converging flows surrounded by a ring of horizontal divergence (consistent with Langfellner et al. 2016). As was also described by Langfellner et al. (2016), the magnetic field distribution is o set in the retrograde direction relative to the flow pattern.

The average preemergence flow measured here is elongated in the east-west direction. We thus assume that emergence locations are distributed relative to the centers of the convergence features with a probability distribution that is elongated in the east-west direction. In particular, we assume that the o sets (*δx* and *δy*) of the synthetic emergence locations follow Gaussian distributions. The distribution for *δx* has a mean of *μ*_*x*_ = 14 pix and a standard deviation of *σ*_*x*_ = 12 pix. The distribution for *δy* has a mean of zero and standard deviation of *σ*_*y*_ = 4 pix.

These values are selected by hand so that the model produces (as we show below) a pattern of flows that is similar to the observations. Figure 5 shows the resulting 2D probability distribution.

We then selected a single convergence feature in each QS map at *t* = −13.6 h. For each of these convergence regions, we generated ten synthetic emergence locations by drawing locations (*δx*, *δ*y) from the probability distribution described in the previous paragraph. These emergence locations were then used to produce an average flow map. The use of ten emergence locations per QS map helps to reduce the noise in the resulting average flow map.

**Fig. 6.**
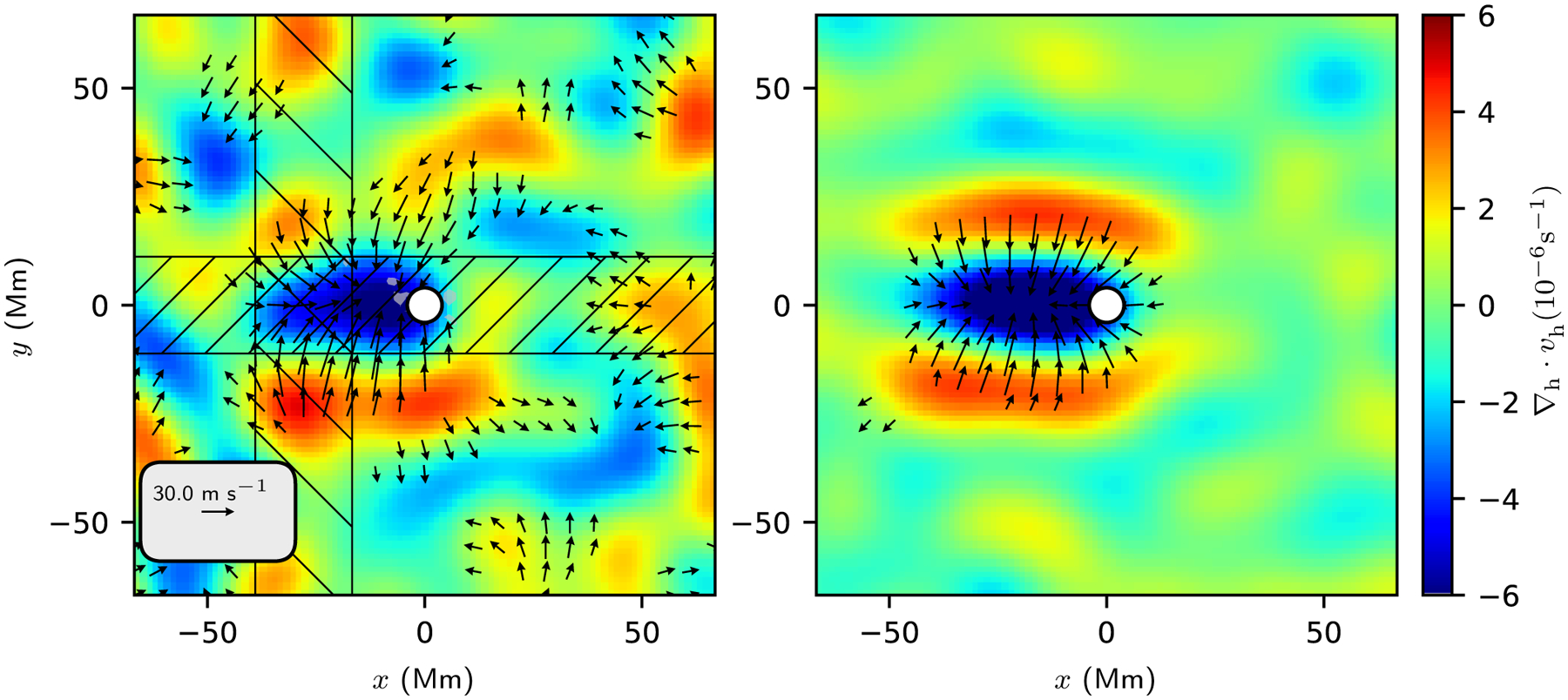
Comparison between the average over emerging active regions at *t* = −13.6 h (*left*) and the model using the probability distribution of synthetic emergence locations from Fig. 5. In both panels, the color in the background shows the horizontal divergence and the black arrows show the horizontal flows. The emergence location is shown by the white circle. Flows of less than 15 ms^−1^ are not shown. The model reproduces the east-west elongated converging flow seen in the observations. Unlike the observations, the model shows a retrograde flow at the emergence location.

Figure 6 compares the measured preemergence flow at 13.6 h before emergence with the model obtained by averaging over the flow fields that are associated with the synthetic emergence locations described in the previous paragraph. The model shows a lower noise level than the observations due to the choice of ten synthetic emergence locations per quiet-Sun control region. The model flow otherwise reproduces many of the qualitative properties of the observations. The converging flow is elongated in the east-west direction (this is a consequence of the choice *σ*_*x*_ > *σ*_*y*_) and is located to the east (retrograde direction) of the emergence location (this is a consequence of the choice of *μ*_*x*_ > 0). Like the observations, the model also shows regions of horizontal divergence to the north and south of the converging flow; these diverging regions are a caused by the diverging flows seen around the averaged feature (Fig. 5).

**Fig. 7.**
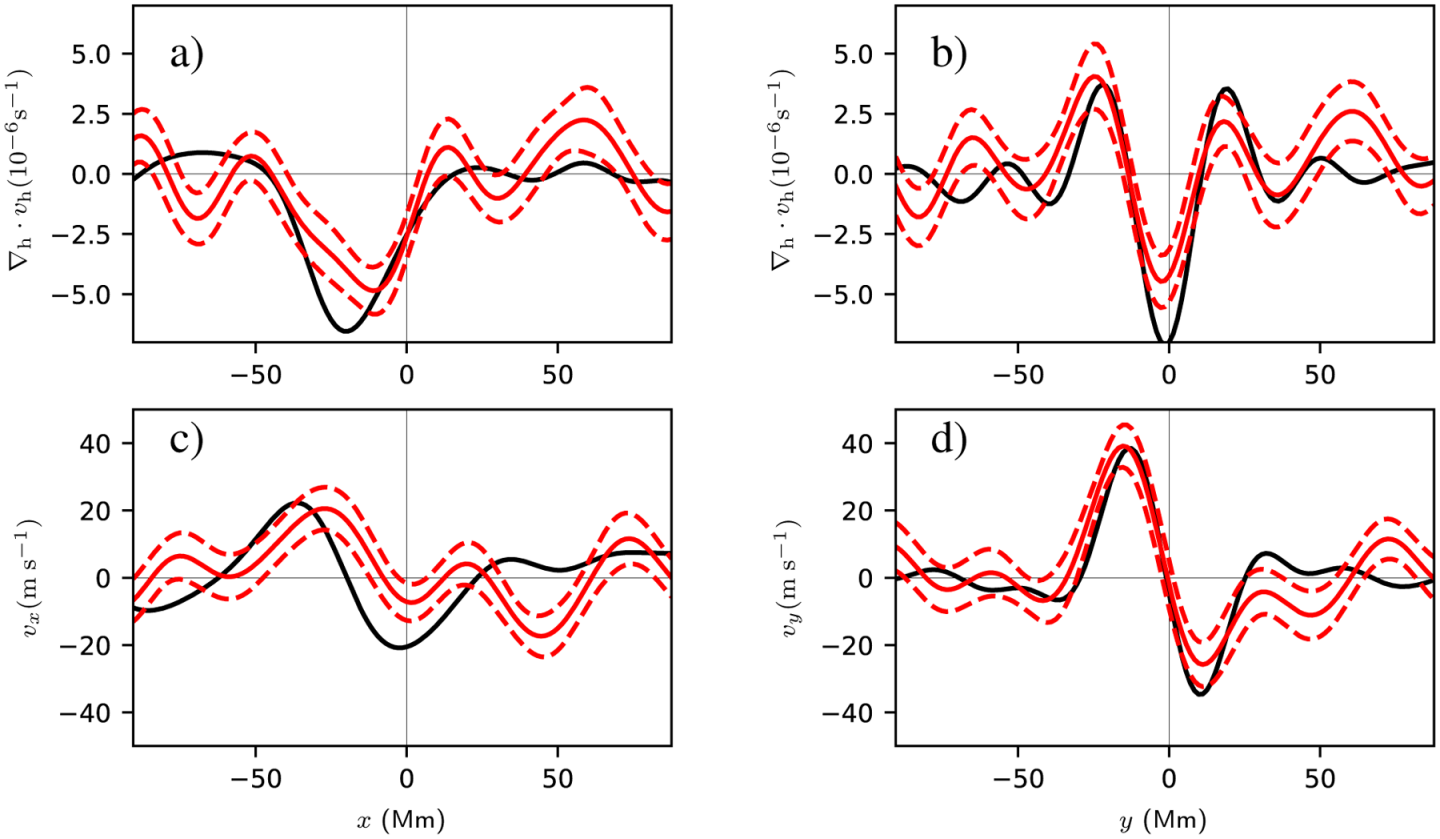
Cuts through the divergence (*top row*), *x* component of the velocity (*bottom left*), and *y* component of the velocity (*bottom right*). The red lines show these quantities for averaging over ARs at *t* = −13.6 h (as in Fig. 4), and the black lines show the corresponding quantities as predicted by the model described in this section. In all panels the divergence or velocity has been averaged in a strip of width 11.1 Mm perpendicular to direction of the cut. The model is a reasonable qualitative explanation of the divergence and *y* component of velocity. For both the observations and the model, the *x* component of the flow is weaker than the *y* component.

Figure 7 shows slices through the observed and modeled flows. As seen in Fig. 6, the model reasonably matches the observations for the horizontal divergence and the north-south flow. The situation is less clear for *v*_*x*_, which is a weaker flow.

## 5. Summary and conclusions

We have shown that helioseismic holography and local correlation tracking provide very similar estimates of the surface flows preceding the emergence of active regions. This comparison gives us confidence that the flows are physically meaningful rather than measurement artifacts. We then showed that in the day before emergence, active region emergence is on average preceded by an east-west elongated converging flow of amplitude about 40 ms^−1^ that is located to the east (retrograde direction) from the emergence location.

We used a simple model based on constructing synthetic emergence locations in the quiet-Sun control regions. We showed that a simple model that assumes that emergence locations are related to supergranulation-scale converging flow regions reproduces the main features of the observations at *t* = −13.6 h. We do not expect that the model presented here is unique in its ability to produce preemergence flows that are similar to the observations. For example, it may be possible to produce models in which the emergence locations are in some way related to supergranulation-scale diverging flows rather than converging flows. The connection between the locations of supergranulation centers and supergranulation-scale convergence regions is only statistical in nature (Langfellner et al. 2018), so that there is a distinction between models based on convergence centers and supergranule centers. We also note that the model presented here is the simplest model that we found that provides a qualitative match to the observations. The general agreement of the model presented here with the observations suggests an interaction between rising flux concentrations and the supergranulation pattern during the emergence process.

Langfellner et al. (2016) showed that the vertical magnetic field is stronger on the prograde side of quiet-Sun supergranulation-scale divergence features and used a simple cork model to demonstrate that this east-west asymmetry is a consequence of horizontal advection and the wave-like behavior of the supergranulation. We speculate that the wave-like behavior of the supergranulation pattern is an important ingredient in modeling the preemergence time evolution presented here.

## Figures and Tables

**Fig. C.1.**
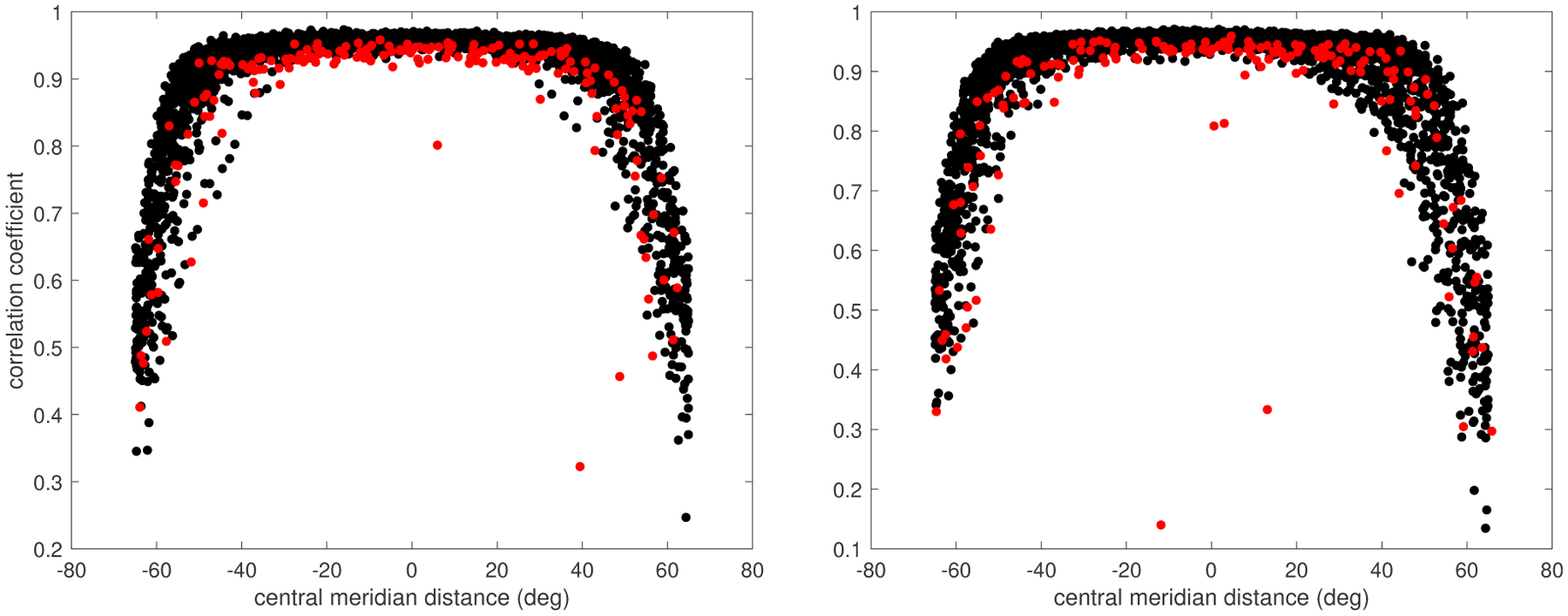
Correlation coefficients for *v*_*x*_ and $v_{x}^{\text{lct}}$ for quiet Sun (*left*) and emerging active regions (*right*) as a function of central meridian distance. Points with a duty cycle above (below) 0.9 are shown in black (red). As described in Sect. 2.1 and Appendix B, large-scale flows were removed by fitting second-order polynomials in *x* and *y*.

## Appendix A: Quiet-Sun measurements

Figure A.1 shows the average flow maps for the quiet-Sun control regions. The quiet-Sun control regions are chosen so that they have the same disk positions as the emerging active regions. The selection procedure is described in detail by Schunker et al. (2016). The average flow maps shows only supergranulation-scale noise.

## Appendix B: Local correlation tracking

The input data for the local correlation tracking (LCT) of the granulation procedure are data cubes of the continuum intensity of length 6.825 h (547 images, same as for the helioseismology analysis). The images were remapped using a Postel projection with the same remapping center as for the Dopplergrams, but with a map scale of 0.348 Mm and using 1024 × 1024 grid points (these maps thus cover only one quarter of the area on the Sun compared to the helioseismology maps). Following the method of Löptien et al. (2016), we applied the Fourier local correlation tracking (FLCT) code (Welsch et al. 2004; Fisher & Welsch 2008) with sigma=6 pix (sigma describes the effective spatial resolution in the resulting flow maps) to estimate horizontal flows at the surface by tracking the motion of granules in the HMI continuum intensity images. We computed flow maps for every pair of consecutive intensity images (45 s cadence) and then averaged all of the flow maps in time to compute a single map for each data cube. We used an iterative outlier rejection algorithm to remove the occasional very strong flows returned by the FLCT code: outliers of more than four standard deviations away from the median were replaced by the average of the flows at the immediately adjacent pixels. As for the case of the helioseismic holography maps, we fit and removed a second-order polynomial in the *x* and *y* pixel coordinates from each map. In order to facilitate the comparison between the LCT flows and the helioseismology, we smoothed the LCT flow maps by 36 pixels (about 12.5 Mm) in the direction of the flow and 24 pixels (about 8.3 Mm) in the perpendicular direction. These smoothing parameters were selected to maximize the correlation between the LCT and the helioseismology maps. The flows resulting from this process were labeled $v_{x}^{\text{lct}}$ and $v_{y}^{\text{lct}}$. Within about 50° of disk center and when the duty cycle of the observations was not too low, the *v*_h_ and $v_{\text{h}}^{\text{lct}}$ flows maps were highly correlated (correlation coefficients typically about 0.96, see Appendix C). Strong correlations between the near-surface flows inferred from helioseismology and LCT have been reported before (e.g., De Rosa et al. 2000; Švanda et al. 2013).

## Appendix C: Comparison of horizontal flows from helioseismic holography and LCT

Figure C.1 shows the correlation coefficient between the quiet-Sun *v*_*x*_ (Sect. 2.1) and $v_{x}^{\text{lct}}$ maps (Appendix B) for each of the emerging active regions and quiet-Sun control regions in the database of Schunker et al. (2016). The overall pattern is much the same for emerging active regions (left panel) and quiet-Sun control regions (right panel). The correlation coefficients are near 0.96 when near disk center and when the duty cycle is reasonable. Beginning at about a central meridian distance of 4°, the correlation coefficient drops with distance from the central meridian; this dropoff steepens sharply at about 50°. We therefore chose a cutoff of 50° as the cutoff in central meridian distance for the regions studied here. Flow maps made when the duty cycle was less than 90% (red points in Fig. C.1) sometimes showed a weak correlation between *v*_*x*_ and $v_{x}^{\text{lct}}$ and were dropped from the averages shown in this paper.

## Appendix D: Identifying supergranulation-scale convergence regions

We used the following procedure to identify the centers of supergranulation-scale converging flows in maps of out-minus-in travel-time differences *τ*_oi_: smooth the maps of *τ*_oi_ with a Gaussian with *σ* = 9.73 Mm (7 pixels); make a list of all the local maxima that have a value greater than 2 s; reject local maxima that are closer than 120 Mm to a pixel where the smoothed (Gaussian *σ* = 5 pix) unsigned field exceeds 120 G; for each entry in the list, find all other entries in the list that are located within 11.12 Mm (8 pixels) and remove from consideration all convergence centers that have a weaker smoothed *τ*_oi_. Finally, select the convergence center that is closest to the center of the map. To obtain the average flow shown in Fig. 5, we used all convergence centers for each map instead of only the convergence center that was closest to the center map.
